# Induction treatment in high-grade B-cell lymphoma with a concurrent *MYC* and *BCL2* and/or *BCL6* rearrangement: a systematic review and meta-analysis

**DOI:** 10.3389/fonc.2023.1188478

**Published:** 2023-07-20

**Authors:** Vanja Zeremski, Siegfried Kropf, Michael Koehler, Niklas Gebauer, Ellen D. McPhail, Thomas Habermann, Francesca Schieppati, Dimitrios Mougiakakos

**Affiliations:** ^1^ Department of Hematology and Oncology, Medical Center, Otto-von-Guericke University Magdeburg, Magdeburg, Germany; ^2^ Department for Biometry and Medical Informatics, Otto-von-Guericke University Magdeburg, Magdeburg, Germany; ^3^ Specialty Practice for Psycho-Oncology, Magdeburg, Germany; ^4^ Department of Hematology and Oncology, University Hospital of Schleswig-Holstein, Luebeck, Germany; ^5^ Department of Laboratory Medicine and Pathology, Mayo Clinic, Rochester, MN, United States; ^6^ Division of Hematology, Mayo Clinic, Rochester, MN, United States; ^7^ Hematology, ASST Spedali Civili di Brescia, Lombardy, Italy

**Keywords:** high-grade B cell lymphoma, double-hit lymphoma (DHL), triple-hit lymphoma (THL), induction treatment, meta-analysis

## Abstract

**Background and aim:**

High-grade B cell lymphomas with concomitant *MYC* and *BCL2* and/or *BCL6* rearrangements (HGBCL-DH/TH) have a poor prognosis when treated with the standard R-CHOP-like chemoimmunotherapy protocol. Whether this can be improved using intensified regimens is still under debate. However, due to the rarity of HGBCL-DH/TH there are no prospective, randomized controlled trials (RCT) available. Thus, with this systematic review and meta-analysis we attempted to compare survival in HGBCL-DH/TH patients receiving intensified vs. R-CHOP(-like) regimens.

**Methods:**

The PubMed and Web of Science databases were searched for original studies reporting on first-line treatment in HGBCL-DH/TH patients from 08/2014 until 04/2022. Studies with only localized stage disease, ≤10 patients, single-arm, non-full peer-reviewed publications, and preclinical studies were excluded. The quality of literature and the risk of bias was assessed using the Methodological Index for Non-Randomized Studies (MINORS) and National Heart, Lung, and Blood Institute (NHLBI) Quality Assessment Tool for Observational Cohort and Cross-Sectional Studies. Random-effect models were used to compare R-CHOP-(like) and intensified regimens regarding 2-year overall survival (2y-OS) and 2-year progression-free survival (2y-PFS).

**Results:**

Altogether, 11 retrospective studies, but no RCT, with 891 patients were included. Only four studies were of good quality based on aforementioned criteria. Intensified treatment could improve 2y-OS (hazard ratio [HR]=0.78 [95% confidence interval [CI] 0.63-0.96]; p=0.02) as well as 2y-PFS (HR=0.66 [95% CI 0.44-0.99]; p=0.045).

**Conclusions:**

This meta-analysis indicates that intensified regimens could possibly improve 2y-OS and 2y-PFS in HGBCL-DH/TH patients. However, the significance of these results is mainly limited by data quality, data robustness, and its retrospective nature. There is still a need for innovative controlled clinical trials in this difficult to treat patient population.

**Systematic review registration:**

https://www.crd.york.ac.uk/prospero, identifier CRD42022313234.

## Introduction

Large B cell lymphomas (LBCLs) represent a rather heterogeneous group of B cell-derived entities (1). The underlying genetic, morphologic, and clinical features of LBCLs can vary substantially translating into different outcomes. More than 20 years ago it was suggested that LBCLs harboring *MYC*, *BCL2*, and/or *BCL6* translocation (double-hit [DH] or triple-hit [TH] lymphoma) fare poorly under standard-intensity chemotherapeutic regimen (2). It was however not until 2017 that this subgroup was introduced as a separate entity and defined as “high-grade B-cell lymphoma with *MYC* and *BCL2* and/or *BCL6* rearrangements” (HGBCL-DH/TH) in the WHO classification (3). Most recently, cases with *MYC*/*BCL6* rearrangement were separated from this group due to the divergent mechanisms of pathogenesis (1).

HGBCL-DH/TH patients frequently present with high-risk features including advanced stage, a high International Prognostic Index (IPI) score, and ≥1 extranodal localization. Furthermore, central nervous system involvement is common (4–8). Earlier works have repeatedly confirmed that HGBCL-DH/TH patients have inferior outcome following standard DLBCL treatment (i.e., R-CHOP), especially in advanced stage (9–12). Consequently, intensified regimens (i.e., DA-EPOCH-R, R-CODOX-M/IVAC, R-hyperCVAD, and GMALL protocol) have been introduced and are currently widely used in first-line setting (13–16). In fact, even adoptive CAR T cells therapy is currently being evaluated as a frontline approach in this highly vulnerable patient population (17). However, data addressing the significance of an intensified first-line therapy have been scarce and rather disputable. Several studies implied that intensified regimens could improve progression-free survival (PFS) (7, 18); yet benefit in terms of overall survival (OS) was rarely reported. Recently, we retrospectively analyzed a large, multi-center HGBCL-DH/TH cohort of 259 patients and could not identify a significant advantage of employing intensified regimens over R-CHOP(-like) regimens (neither for PFS nor for OS) (19).

Thus, we decided to re-evaluate this issue and to possibly gain additional insights that would help guide the treatment of this difficult-to-treat population. With this aim, we performed a meta-analysis of recently published studies and compared survival outcome of intensified regimens to R-CHOP(-like) strategies in newly diagnosed HGBCL-DH/TH patients.

## Methods

This systematic review and meta-analysis was preformed following the Preferred Reporting Items for Systematic Reviews and Meta-Analyses (PRISMA) guidelines (20). The protocol was registered at PROSPERO International Prospective Register of Systematic Reviews (CRD 42022313234).

### Selection criteria and search strategy

The Population, Intervention, Control, Outcome, Study (PICOS) approach was used to define the inclusion criteria (**Table 1**) (21). Eligible studies were included in the analysis if treatment related outcomes were reported. The primary endpoint in our study was 2-year OS (2y-OS) of patient groups receiving different induction regimens (intensified regimen vs. R-CHOP[-like]). Furthermore, we compared 2-year PFS (2y-PFS) between these 2 groups. In case of eligible studies not reporting on treatment related outcome, the authors were contacted in order to obtain missing data necessary for analysis. The exclusion criteria were also reported in **Table 1**. Literature search for studies published from 08/2014 until 04/2022 was carried out using the PubMed and Web of Science databases. The following keywords were used, with the use of wildcard characters to account for variations in spelling and plurals: “*MYC/BCL2*” OR “*MYC/BCL6*” AND “lymphoma”, “double-hit” OR “triple-hit” AND “lymphoma”. Two of the authors independently performed the screening and identified studies, data selection, and data extraction. Disagreements were resolved by a consensus-based discussion. For studies with multiple publications or overlapping patient cohorts, the most complete dataset amongst all available publications was used.

**Table 1:** Inclusion and exclusion criteria for selection of the articles.

PICOS inclusion criteria	
Population	Newly diagnosed HGBCL patients with concurrent *MYC* and *BCL2* and/or *BCL6* rearrangement (according to WHO 2017) aged ≥18 years
Intervention	Intensified front-line treatment (i.e. DA-EPOCH-R, R-CHOEP, R-Hyper-CVAD, R-CODOX-M/IVAC, B-ALL protocol, R-CHOP with upfront autologous SCT)
Control	Standard front-line treatment (R-CHOP[-like]: R-CHOP and its modified versions [i.e. R-miniCHOP, R-CHOP with lenalidomide or ibrutinib])
Outcome	2-years overall survival and/or progression-free survival
Study design	Randomized clinical trials, retrospective trials and case series written in English language and published between August 2014 and April 2022 (including e-publications available ahead of print)
Exclusion criteria	
	Studies ≤10 patients, single-arm studies, reviews, preclinical trials, case reports, abstracts, posters

PICOS, Population, Intervention, Control, Outcome, Study; HGBCL, high-grade B-cell lymphoma; SCT, Stem cell transplantation.

### Study quality assessment

Methodological Index for Non-Randomized Studies (MINORS) was used to assess the quality of included observational studies (22). The MINORS consists of 12 indexes: 1) a clearly stated aim; 2) inclusion of consecutive patients; 3) prospective collection of data; 4) endpoints appropriate to the aim of the study; 5) unbiased assessment of the study endpoint(s); 6) a follow-up period appropriate to the aim of the study; 7) loss to follow-up less than 5%; 8) prospective calculation of the study size; 9) an adequate control group; 10) contemporary groups (control and studied group should be managed during the same time period, no historical comparison); 11) baseline equivalence of groups and 12) an adequate statistical analysis. The items were scored 0 if not reported; 1 when reported but inadequate; and 2 when reported and adequate. Studies were considered as high quality if the total score was ≥17, medium quality if the total score was 9-16, and low quality if the total score was <9 (22). In addition, the NHLBI Quality Assessment Tool for Observational Cohort and Cross-Sectional Studies (NHLBI, National Heart, Lung, and Blood Institute) was used (23). NHLBI developed a set of tailored quality assessment tools to assist reviewers in focusing on concepts that are key to a study’s internal validity. We used the study rating tool on the range of items included in each tool to judge each study to be of “good,” “fair,” or “poor” quality. In general terms, a “good” study has the least risk of bias, and results are considered to be valid. A “fair” study is susceptible to some bias deemed not sufficient to invalidate its results. The fair quality category is likely to be broad, so studies with this rating will vary in their strengths and weaknesses. A “poor” rating indicates significant risk of bias (23). The study quality was assessed by two authors.

### Data analysis

All statistical analyses were performed using SPSS software, version 28 (IBM Statistics, Armonk, NY, USA). Heterogeneity between studies was assessed using I^2^ statistics, with I^2^ above 50% being considered as an indicator for distinct heterogeneity. As large heterogeneity among the studies was observed particularly for PFS, the random-effects model has been applied consistently for all analyses. The hazard ratio (HR) with a corresponding 95% confidence interval (95% CI) for OS and PFS was utilized to compare the prognostic survival. HR and 95% CI were directly extracted from the Cox proportional hazards models used for the univariate analyses of the original studies. When it was not possible to obtain these values from the original studies, we estimated them from survival curves (where possible) using the methods described by Parmar et al. (24) or we derived them from the reported estimates and CIs for 2yOS or 2yPFS in the two treatment arms via normal approximation. HR less than 1.0 indicated an advantage for intensified treatment as compared to standard treatment in terms of improving 2y-OS and 2y-PFS. *p* values ≤0.05 were considered statistically significant. Potential publication bias was examined using funnel plot and Eggers’ test. Robustness of results was assessed by iterative omission of single studies from the analysis (so-called leave-one-out analysis).

## Results

### Study selection and description of studies

**Figure 1:**
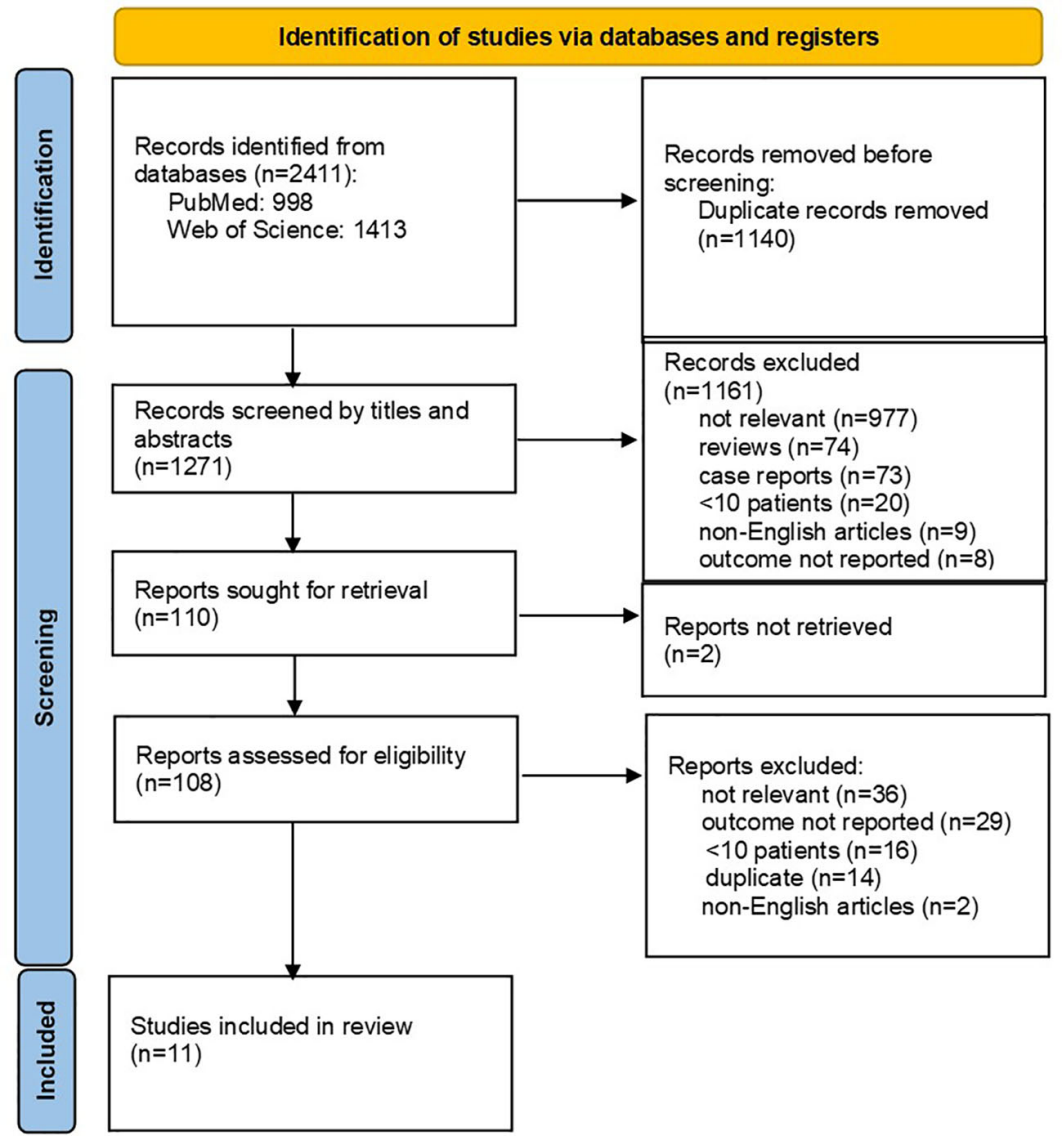
PRISMA flow diagram.

Altogether 2411 records were identified through database searches. After initial screening, 108 full-text articles were further selected for eligibility. The flowchart of the reviews shows the detailed process of selection (**Figure 1**). Finally, 11 relevant studies, comprising a total of 891 HGBCL-DH/TH patients, were included (6, 7, 18, 25–32). We identified no prospective, randomized controlled trials (RCT); all studies included in the meta-analysis were of retrospective design. Three authors provided additional individual patient data on request (26, 27, 29). Characteristics of included studies are outlined in **Table 2**. *MYC* translocation partner was rarely stated, therefore we did not report on this data. Studies conducted exclusively on HGBCL-DH/TH patients with localized stage (n=3) were excluded, in order to avoid selection bias. Eight out of 11 eligible studies were published from 2019 onwards (6, 18, 26–29, 31, 32). Only four studies were primarily designed to compare induction treatment in HGBCL-DH/TH patients (6, 7, 18, 31).

**Table 2:** Characteristics of included studies.

Study	Country	Sample size (*N*)	Participant group (*n*)	Treatment arm, n (%)	age, median (range)	*BCL2* +/- *BCL6* translocation, n (%)	advanced stage	high IPI, n (%)	FU months, median (range)	2y-PFS, %	2y-OS, %
*de Jonge* et al., 2016^§#^	Netherland	26	17	R-CHOP, 10 (58.8) intensified^1^, 7 (41.2)	64.5 (41–80)	8 (80.0) 7 (100)	9 (90.0) 7 (100.0)	na	40.5 (5.6-75.4)	na	36.0 53.6
*Kuenster* et al., 2021^§#^	Germany	47	34	R-CHOP-like^2^, 21 (61.8) intensified^2^, 13 (38.2)	73 (45-82) 60 (35-77)	11 (52.4) 10 (76.9)	10 (47.6) 12 (92.3)	13 (61.9) 9 (69.2)	92 (70.3-113.7)	42.9 23.1	61.9 46.2
*Laude* et al., 2021^#^	France	156	156	R-CHOP-like^3^, 99 (63.5) intensified^3^, 57 (36.5)	66 58	76 (76.8) 46 (80.7)	85 (85.9) 55 (98.2)	69 (75.0) 39 (72.0)	32 (28-39)	40.0 50.7	na
*McPhail* et al., 2019^§#^	USA	100	70	R-CHOP, 32 (45.7) intensified^4^, 38 (54.3)	66 (44-79) 59 (29-83)	25 (83.3) ns, 2 28 (82.4) ns, 4	na	na	28.9 (14.3-43.5)	29.8 53.9	41.5 57.5
*Miyaoka* et al., 2022^§^	Japan	50	21	R-CHOP-like^5^, 15 (71.4) intensified^5^, 6 (28.6)	67 (49-79) 57.5 (39-73)	13 (86.6) 4 (66.7)	10 (66.7) 4 (66.7)	10 (66.7) 1 (16.7)	78 (0-179.3)	na	41.5 57.5
*Petrich* et al., 2014^#^	USA	311	311	R-CHOP, 100 (32.0) intensified^6^, 211 (57.9)	60 (19-87)*	295 (95.0)*	255 (81.0)*	na	23 (1-126)**	na	na
*Schieppati* et al., 2020^§#^	Italy	95	24	R-CHOP, 7 (29.2) intensified^7^, 17 (70.8)	68 (59-88) 62 (27-76)	5 (71.5) 14 (82.3)	6 (85.7) 16 (94.1)	4 (57.1) 11 (64.7)	45 (28.6-58.1)	42.9 73.1	42.9 58.8
*Tisi* et al., 2019	Italy	100	76	R-CHOP^8^, 27 (33.3) intensified^8^ 34 (42.0) DA-EPOCH-R, 15 (19.0)	61 (21-85)*	57 (70.4)*	70 (86.0)*	56 (69.0)*	33**	40 50 64	34 64 66
*Yoshida* et al., 2015^§^	Japan	22	12	R-CHOP, 7 (58.3) intensified, 5^9^ (41.6)	66 (54-88) 58 (46-64)	7 (100.0) 5 (100.0)	6 (75.9) 5 (71.4)	4 (57.1) 4 (80.0)	14 (5.6-22.4)	na	51.4 40.0
*Zhang F*. et al, 2019	China	139	139	R-CHOP, 76 (54.7) intensified, 63^10^ (45.3)	57 (18-81)*	*BCL2*, 109 (78.4)*	54 (71.1) 36 (57.1)	34 (44.8) 42 (66.7)	18 (4-39)	45.4 63.6	47.8 67.4
*Zhang J*. et al. 2020^§#^	China	51	31	R-CHOP-like^11^, 18 (58.1) intensified, 13^11^ (41.9)	56.5 (26-72) 42 (19-68)	ns	13 (72.2) 9 (61.5)	na	16 (10.4-21.5)	na	87.7 58.6

Sample size (N) refers to the total number of patients in each study, whereas the participant group (n) refers to the number of HGBCL-DH/TH patients included in this meta-analysis (i.e. HGBCL-DH/TH patients who received [curative] induction treatment and had reported treatment outcome).

^§^available individual patient data, ^#^including HGBCL-DH/TH arising from low-grade lymphomas, * data for all patients; **follow-up for all living patients.

ns, not specified; na, not applicable.

^1^ intensified regimens: DA-EPOCH-R (dose-adjusted, rituximab, etoposide, prednisone, vincristine, cyclophosphamide and doxorubicin) 71.4%, R-CHOP+up-front autologous stem cell transplantation (SCT) 28.6%; R-DHAP (rituximab, dexamethasone, cisplatin, high-dose cytarabine) 14.2%.

^2^ R-CHOP-like: R-CHOP(rituximab, cyclophosphamide, doxorubicin, vincristine, prednisone), R-miniCHOP.

intensified regimens: GMALL (German multicenter acute lymphoblastic leukemia) protocol 61.5%, R-CHOEP (rituximab, cyclophosphamide, doxorubicin,

vincristine, etoposide, prednisone) 23.1%, DA-EPOCH-R 7.7%, other 7.7%.

^3^ R-CHOP-like not further specified.

intensified regimens: DA-EPCOH-R 24.6%, R-ACVBP (rituximab, doxorubicin, cyclophosphamide, vindesine, bleomycin and prednisone) 28.1%, R-COPADEM (rituximab, cyclophosphamide, vincristine, prednisone, doxorubicin and methotrexate) 47.3%.

^4^ intensified regimens: R-CHOEP 44.7%, R-CODOX-M/IVAC 39.5%, R-hyperCVAD (rituximab, cyclophosphamide, vincristine, doxorubicin and

dexamethasone) 15.8%.

^5^ R-CHOP-like: R-CHOP, R-COP (rituximab, cyclocphosphamid, vincristine, prednisone).

intensified regimens: DA-EPOCH-R 66.7%, R-hyper-CVAD 33.3%, R-CODOX-M 16.7%.

^6^intensified regimens: R-Hyper-CVAD n=65, DA-EPOCH-R n=64, R-CODOX-M/IVAC n=42, R-ICE n=9, other n=10; up-front autologous SCT n=39, up-front

allogeneic SCT n=14.

^7^intensified regimens: DA-EPOCH-R (+/- up-front autologous stem cell transplantation) 58.9%, GMALL 35.3%, R-CHOP+up-front autologous SCT 5.9%.

^8^R-CHOP-like: R-CHOP, R-COMP (R-CHOP with liposomal anthracycline), R-miniCHOP, R-megaCHOP, R-M/VACOP-B (rituximab, methotrexate, etoposide,

doxorubicin, cyclophosphamide, vincristine, prednisone and bleomycin).

intensified regimens: R-CODOX-M/IVAC, GMALL, R-Hyper-CVAD/R-MA (rituximab, cyclophosphamide, vincristine, doxorubicin and dexamethasone/

rituximab,high-dose methotrexate and cytarabine), upfront autologous SCT (accurate treatment distribution not known).

^9^intensified regimens: R-CODOX-M/IVAC (rituximab, cyclophosphamide, doxorubicin, vincristine, methotrexate/ifosfamide, etoposide, high-dose cytarabine)

60.0%, R-hyper-CVAD 20.0%, R-ESHAP (rituximab, etoposide, methylprednisolone, high-dose cytarabine and cisplatin) 20.0%.

^10^intensified regimens: DA-EPOCH-R (100 %).

^11^R-CHOP-like: R-CHOP, R-CHOP + lenalidomide, R-CHOP + HD-MTX.

intensified regimens: DA-EPOCH-R +/- HD-MTX or +/- i.th. MTX (76.9%); R-CODOX-M/IVAC (15.4%), other (7.6%).

### Quality assessment

No standardized assessment tools exist for observational studies. We used the MINORS scale to assess study quality (**Supplementary Table S1**). The final scores for each study ranged from 13 to 19. Overall, the studies included in the meta-analysis were of intermediate reliability. Four studies were considered to be high-quality studies (6, 26, 27, 31). We additionally assessed studies for bias based on the 14 criteria in the NHLBI tool for quality assessment (NHLBI). About a half (54.5%) of the studies were of good quality and showed low risk of bias. All studies lacked the following features: blinded study, exposure reassessment over time, provided sample size justification, power description or variance/effect estimates. Key methodological strengths in the included studies were rare.

### Outcome

**Figure 2:**
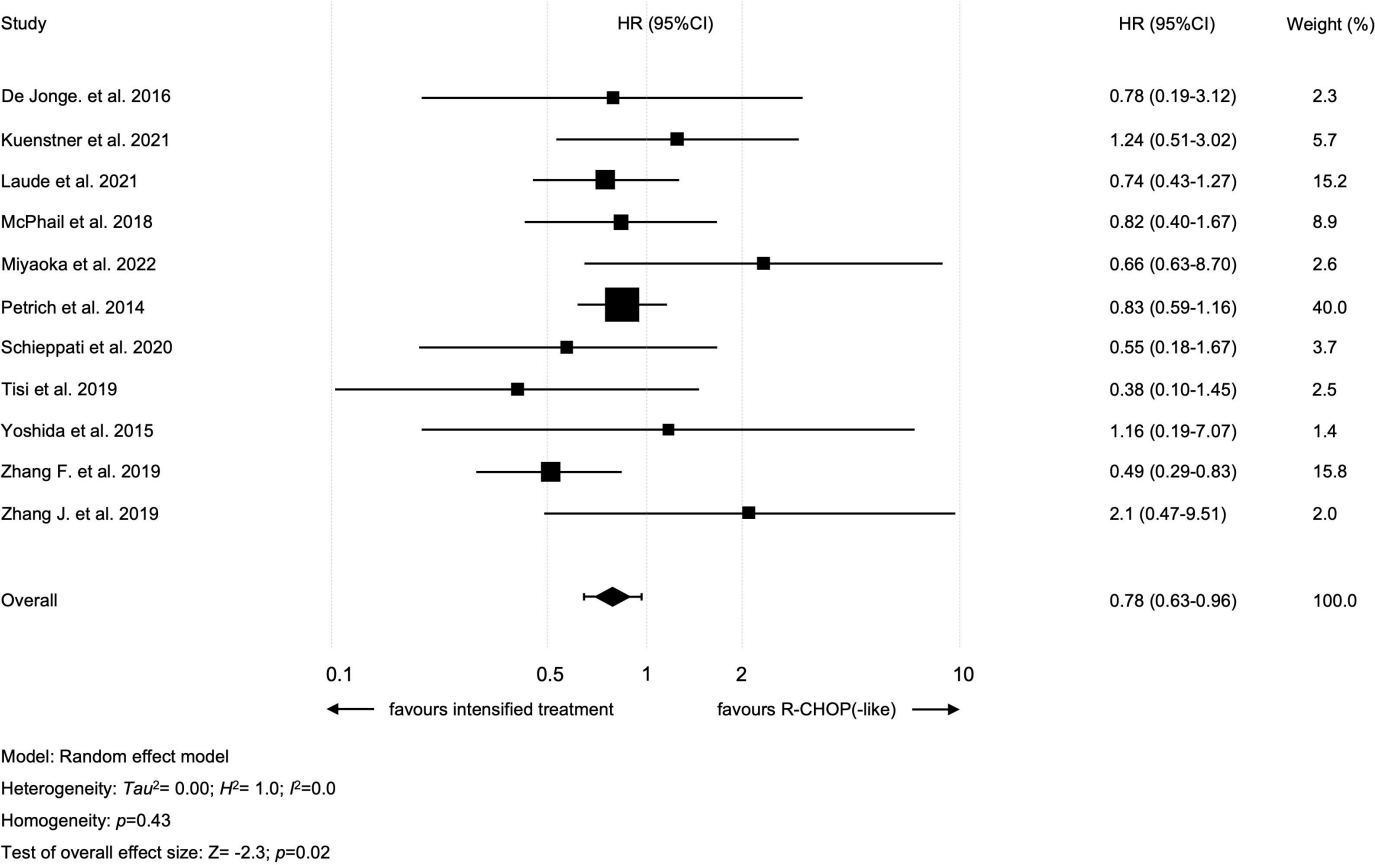
Forest plot of 2-year overall survival.

All studies were analyzed regarding 2y-OS and included 464 patients that received an intensified treatment and 412 with standard R-CHOP-like protocol. The study by Tisi et al., compared R-CHOP to two different treatment groups: i.e., DA-EPOCH-R and “intensive regimens” (R-CODOX-M/IVAC, R-Hyper-CVAD/R-MA, GMALL protocol) (18). Only patients treated within the “intensive regimens” cohort were included in our analysis (as this cohort included more patients than the DA-EPOCH-R group). Intensified treatment resulted in improved 2y-OS in all patients (intensified treatment *vs.* standard treatment: HR=0.78 [95% CI 0.63-0.96]; *p*=0.02; **Figure 2**). There was no heterogeneity among studies (I^2 =^ 0%; *p*=0.43). A sensitivity analysis was performed using the so-called leave-one-out approach in order to evaluate the robustness of the results. The statistically significant combined effect size for the impact of intensified treatment on 2y-OS was found to be lost when omitting one of the following studies: Laude et al., Petrich et al. and Zhang et al. (**Supplementary Table S2**) (6, 7, 31). No publication bias with regard to 2y-OS was evident (*p*=0.11 in Egger’s test; funnel plots are presented in **Supplementary Figure S1**).

**Figure 3:**
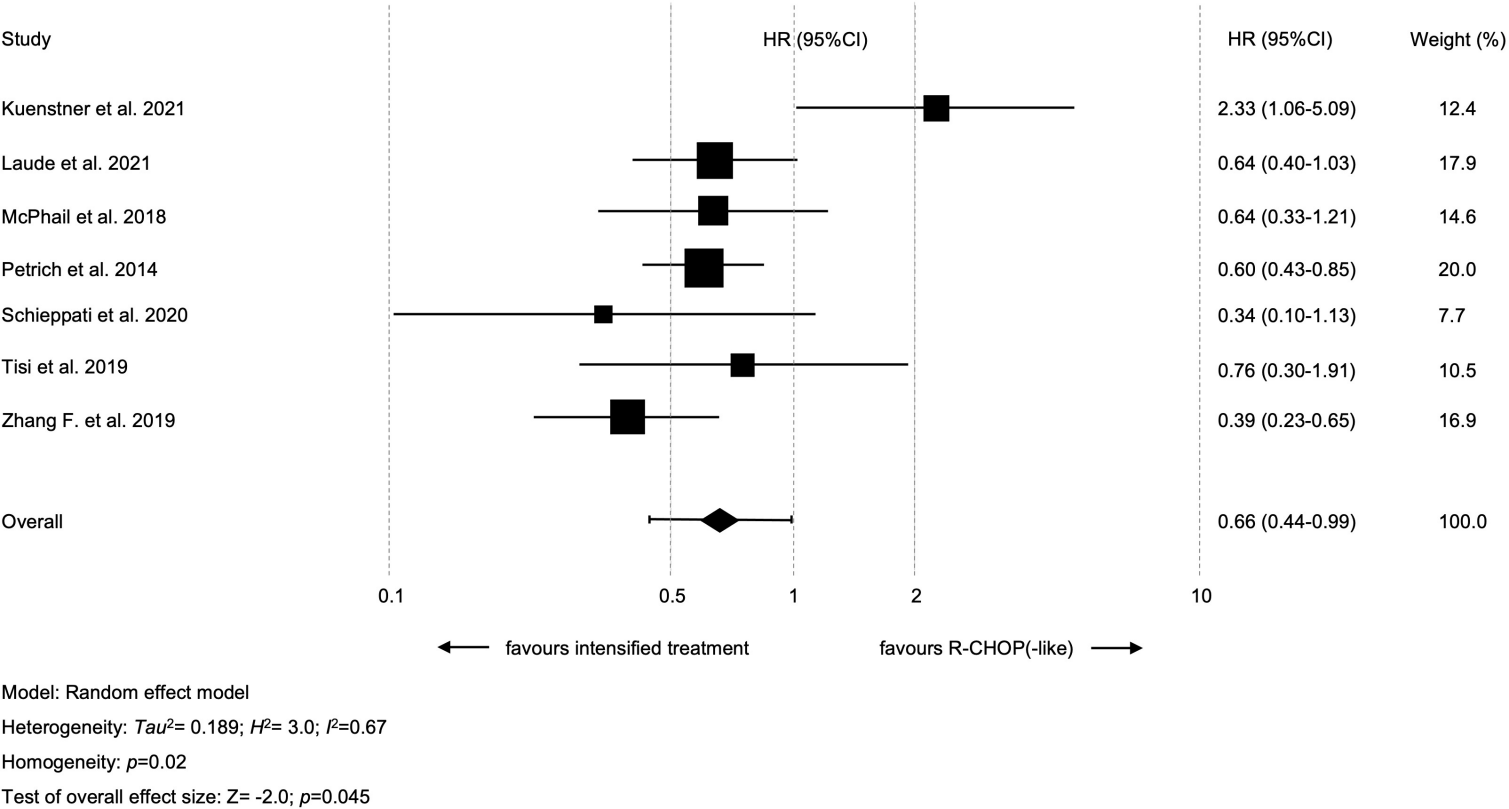
Forest plot of 2-year progression-free survival.

Seven studies with available data for 2y-PFS were included in the meta-analysis; altogether, 358 patients treated with intensified and 325 patients treated with standard treatment. Intensified treatment was shown to result in prolonged 2y-PFS as compared to standard treatment (HR=0.66 [95% CI 0.44-0.99]; *p*=0.045, **Figure 3**). A significant heterogeneity between the results of the individual studies (I^2 =^ 66.7%; *p*=0.02) was noticed. The study by Kuenstner et al. was shown to be the key contributor to this between-study heterogeneity after performing the so-called leave-one-out sensitivity analysis (26). Following exclusion of this study, the combined effect size for the impact of intensified treatment on 2y-PFS was found to be stronger (HR=0.57 [95% CI 0.45-0.7]; *p*<0.01) and there was no longer heterogeneity among studies (I^2 =^ 0%; *p*=0.60) (**Supplementary Table S3**). There were no hints on publication bias regarding 2y-PFS (*p*=0.41 in the Egger’s test; funnel plots are presented in **Supplementary Figure S2**).

### Toxicity

Treatment toxicity was only questioned within two studies (6, 31). As expected, Laude et al. observed significantly higher rates of grade 3/4 hematological toxicities and mucositis in the intensified arm (neutropenia, thrombocytopenia, anemia, and mucositis; all *p=*0.01). Surprisingly, Zhang et al. reported no difference between these standard versus intensified approaches. Also, neutropenia rates were unexpectedly low in patients treated with intensified regimens (20.6%). Due to the small sample size we refrained from further statistical analyses.

## Discussion

This systematic review of published studies from 2014 to 2022, which compared survival rates according to induction treatment in newly diagnosed HGBCL-DH/TH patients, yielded 11 retrospective studies. Only four of them were primarily designed to compare outcome between intensified treatment and R-CHOP(-like) standard protocols.

Regarding OS, previous data has been rather controversial. A few single-arm, prospective studies reported impressive survival rates using various intensified regimens, as follows: 4-year OS of 82% with DA-EPOCH-R, 2y-OS of 76% with R-CODOX-M/R-IVAC and 5-year OS of 83% with Nordic Lymphoma Group protocol (R-CHOP/R-CHOEP combined with high-dose methotrexate and intrathecal liposomal cytarabine) (33–35). Given the rarity of HGBCL-DH/TH, these studies included only a small number of HGBCL-DH/TH patients (n=24 and n<10 patients, respectively). Conversely, retrospective studies reported comparably low survival rates when applying these (or similar) intensified regimens, with only a few of them reporting a 2y-OS of >60% (18, 31), indicating a potential selection bias toward enrollment of healthier patients in prospective studies.

R-CHOP in combination with lenalidomide was prospectively investigated in 82 LBCL patients with *MYC* translocation (also including 24% patients with *MYC* single-hit translocation) within the HOVON-2 trial (36). This resulted in 2y-OS of 73% and 2-year event-free survival (2y-EFS) of 63%. The REMoDL trial, that compared R-CHOP plus bortezomib *vs*. R-CHOP, included 35 HGBCL-DH/TH patients (37). Median OS at 30 months was 58.5% and 38.9%, respectively.

According to this systematic review, and to the best of our knowledge, no prospective trials have directly compared the efficacy of intensified treatment and R-CHOP(-like) so far. A previously published meta-analysis (7), including 11 retrospective trials (published between 2009 and 2014) with altogether 394 patients, did not find any difference in OS between the two approaches (R-CHOP *vs.* R-EPOCH: HR=0.77 [95% CI 0.51-1.13]; *p*=0.19; R-CHOP *vs.* other intensified regimens [R-Hyper-CVAD, R-CODOX-M/IVAC, R-ICE, and other]: HR 0.89 [95%CI 0.62-1.13]; *p*=0.53). Of note, 5 of the included studies have only reported preliminary results in abstract format and not the final study data. Howlett et al. also included double-expressors (DEL) as well as LBCLs with amplifications and/or extra copies of *MYC*, *BCL2* and *BCL6* in their analysis (38). These, however, have to be distinguished from HGBCL-DH/TH as their prognosis does not seem to differ significantly from DLBCL (NOS) (11, 29, 39, 40). Furthermore, DEL and HGBCL-DH/TH are considered to have different underlying biology. DEL arise from “activated” B-cells in contrast to HGBCL-DH/TH with germinal B-center origin (10, 41).

Our current meta-analysis suggests that 2y-OS can be improved using intensified regimes (HR=0.78; *p*=0.02). Still, this needs to be interpreted with caution, as the analysis was found to be insufficiently robust. So-called leave-one-out analysis with any of the three largest trials (Laude et al. n=156, Petrich et al. n=311, Zhang F. et al. n=139) was associated with loss of statistical significance (6, 7, 31).

Regarding PFS, previously published data are also rather contradictory. The earlier mentioned meta-analysis by Howlett et al. demonstrated prolonged PFS when using R-EPOCH (HR=0.66 [95% CI 0.44-0.96]; *p*=0.03). The use of other intensified regimens, however, did not lead to statistically significant improvement of PFS (HR=0.74 [95% CI 0.51-1.05]; *p*=0.09) (38). In our recently published multi-center analysis on HGBCL-DH/TH patients (also including 7 trials with 209 patients from this meta-analysis) neither 2y-OS nor 2y-PFS was shown to be improved with regimens other than R-CHOP(-like) (R-CHOP[-like] *vs.* intensified treatment rates for 2y-OS were 54.2% *vs*. 55.2% [*p*=0.87] and 2yPFS 44.4% *vs*. 48.4% [*p*=0.63], respectively) (19). A subgroup analysis of different intensified regimens (i.e., R-EPOCH *vs.* other treatments) was not carried out. These results were in line with recently published data on 154 HGBCL-DH/TH patients (42). Magnusson et al. reported 4-year OS rates of 54.5% and 49.6% in patients treated with R-CHOP and R-EPOCH, respectively. However, the present meta-analysis did demonstrate an improved 2y-PFS using intensified regimens over R-CHOP(-like) (HR=0.66; *p*=0.045). Interestingly, one study included in this meta-analysis showed improved 2y-PFS with R-CHOP(-like) protocols (26). This is possibly explained by selection bias as these patients were older (median age 73 *vs*. 60 years), had less frequently advanced stage disease (47.6% *vs*. 92.3%), and less often concomitant *BCL2* translocation (52.4% *vs.* 76.9%), signifying an enrichment in the pathogenetically and clinically divergent MYC/*BCL6* rearranged subgroup. In fact, excluding this study from the meta-analysis, enhanced the cumulative effect (HR=0.56; *p*<0.01).

When comparing induction regimens in HGBCL-DH/TH patients, it needs to be mentioned that there is a relevant heterogeneity among this group. Namely, localized stage HGBCL-DH/TH result in high 2y-OS >80% using R-CHOP (with/without consolidative radiation) (43, 44), which is comparable to outcome in DLBCL patients (45). Thus, intensified treatment does not seem to be required in these patients. On the other hand, transformed HGBCL-DH/TH (with a prior history of low grade lymphoma) seem to perform poorly comparing to *de novo* HGBCL-DH/TH.

McPhail et al. reported a median OS of 10.8 months and 22 months in patients with transformed and *de novo* HGBCL-DH/TH, respectively (27). Conversely, Li et al. failed to reproduce these results (46). It is to be mentioned that a number studies do not evaluate/report whether prior low-grade lymphoma was present or not, leaving this issue still unresolved.

HGBCL-DH/TH also encompasses both large cell and high grade morphology, and in some studies high grade morphology shows an association with poorer outcome (27). In addition, the prognostic role of *BCL6* rearrangement is not clear. There are data that suggest that patients with concomitant *MYC*/*BCL6* rearrangement (in the absence of a *BCL2* rearrangement) have a better survival as compared to patients harboring a *MYC/BCL2* rearrangement (47). In fact, gene expression profile and mutational spectra in *MYC*/*BCL6* were shown to differ noticeably from *MYC*/*BCL2* lymphomas (26, 48). Consequently, *MYC/BCL6* LBCLs are now excluded from the HGBCL-DH entity, according to the recently revised 2022 WHO classification (1). Depending on the morphological features they are classified as DLBCLs NOS or HGBLs NOS. The recently updated International Consensus Classification also redefined the term of HGBCL-DH. It now comprises two groups: HGBCL with *MYC/BCL2* rearrangements (with or without *BCL6* rearrangement) and a new provisional entity, HBGBL with *MYC/BCL6* rearrangements (49). Finally, the prognostic significance of the *MYC* translocation partner (immunoglobulin [Ig] *vs*. non-Ig) is not clarified yet. Two large trials (Lunenburg Lymphoma Biomarker Consortium and GELY/LYSA trial) showed that adverse prognosis of *MYC* rearrangement is confined solely to *MYC*/Ig translocation (50, 51). However, several studies failed to show a difference in outcome between *MYC*/Ig and *MYC*/non-Ig rearranged cases (27, 46, 52). The heterogeneity among HGBCL-DH/TH patients possibly explains discordant outcomes in previously published studies.

Another important issue that needs to be considered, is the high heterogeneity among “intensified regimens”. This term includes basically all regimens beyond R-CHOP(-like) (**Table 2**). Some of the previously published studies suggest that treatment outcome can significantly vary among intensified regimens (5, 29). In fact, “more intensified regimens” (i.e. GMALL protocol, R-CODOX-M/IVAC) yield poorer survival rates comparing to DA-EPOCH-R, possibly due to increased toxicity. Further treatment escalation, in terms of consolidative autologous stem cell transplantation, also failed to improve survival rates, especially after intensified induction (53, 54).

The results of our meta-analysis suggest that intensified induction improves 2y-PFS and possibly 2y-OS. One could however argue, whether a superior 2y-PFS suffices to justify the use of an intensified induction. In LBCLs, 2-years 2y-EFS was shown to be a robust parameter for long-term survival (55). Whether this also applies to HGBCL-DH/TH remains to be elucidated. In order to significantly improve OS, new consolidation strategies may be a reasonable approach. Actually, first results of upfront use of CAR T cells in high-risk DLBCL, including 16 HGBCL patients, showed promising results (estimated 12 months OS was 91%) (17). Another phase II study explored blinatumomab consolidation after R-CHOP treatment in high-risk DLBCL (12 HGBCL-DH/TH patients). A notable proportion of patients (i.e., 7/8) with persistent disease after induction (either partial remission or stable disease) did achieve a complete remission after treatment with blinatumomab (56).

However, there are some limitations of this meta-analysis and the applicability of its conclusions. Firstly, the data presented here are derived from retrospective studies and subject to potential sources of bias inherent to this methodology, including missing data, and a non-uniform follow-up. In part, the small size of included studies (<50 patients in six studies) with wide 95% CIs of the estimated HRs in some of them may influenced the reliability of the results. Then, the issue of treatment-related toxicity could not be addressed here, given the scarcity of reported data. Furthermore, most of the studies had a more exploratory design. Evaluating whether there was adequate statistical analysis or an adequate control group was therefore challenging in terms of bias assessment.

In summary, this meta-analysis represents a comprehensive review of the treatment of HGBCL-DH/TH patients. Given the rarity of this entity there is obviously a lack of large high-quality studies. In the absence of a more robust data set, this meta-analysis provides the rationale for using intensified induction protocols for appropriately selected advances stage patients or to preferentially treat them within clinical trials. Moreover, each patient should be counseled on the risks and benefits of such treatment intensification including the limitation of the available data. However, to definitely clarify the question of the optimal induction in HGBCL-DH/TH patients, prospective, randomized trials are promptly needed.

## Data availability statement

The original contributions presented in the study are included in the article/**Supplementary Material**. Further inquiries can be directed to the corresponding author.

## Author contributions

VZ designed and performed the research, interpreted data and wrote the manuscript. SK analyzed and interpreted data. MK performed the research, interpreted data and reviewed the manuscript. NG, EM and TH reviewed the manuscript. DM interpreted data and wrote the manuscript. All authors contributed to the article and approved the submitted version.
