# Supplementary material for: Induction treatment in high-grade B-cell lymphoma with a concurrent MYC and BCL2 and/or BCL6 rearrangement: a systematic review and meta-analysis
Source: Front Oncol. 2023 Jul 20;13:1188478. doi: 10.3389/fonc.2023.1188478 (PMC10399221; doi:10.3389/fonc.2023.1188478)

**Supplementary data**

**Supplementary Table 1**. Studies quality assessment

|  | A clearly stated aim | Inclusion of consecutive patients | Prospective collection of data | Endpoints appropriate to the aim of the study | Unbiased assessment of the study endpoint | Follow-up period appropriate to the aim of the study | Loss to follow up less than 5% | Prospective calculation of the study size | An adequate control group | Contemporary groups | Baseline equivalence of groups | Adequate statistical analyses | Total |
| --- | --- | --- | --- | --- | --- | --- | --- | --- | --- | --- | --- | --- | --- |
| de Jonge 2016 | 1 | 2 | 0 | 1 | 2 | 2 | 2 | 0 | 2 | 2 | 1 | 0 | 15 |
| Kuenster 2021 | 2 | 2 | 0 | 1 | 2 | 2 | 2 | 0 | 2 | 2 | 1 | 1 | 17 |
| Laude 2021 | 2 | 2 | 0 | 1 | 2 | 2 | 2 | 0 | 2 | 2 | 2 | 2 | 19 |
| McPhail 2019 | 2 | 2 | 0 | 1 | 2 | 2 | 2 | 0 | 2 | 2 | 1 | 2 | 18 |
| Miyaoka 2022 | 1 | 1 | 0 | 1 | 2 | 2 | 2 | 0 | 2 | 0 | 1 | 2 | 14 |
| Petrich 2014 | 2 | 2 | 0 | 1 | 2 | 1 | 2 | 0 | 2 | 2 | 0 | 2 | 16 |
| Schieppati 2020 | 1 | 2 | 0 | 1 | 2 | 2 | 2 | 0 | 2 | 1 | 1 | 1 | 15 |
| Tisi 2019 | 2 | 1 | 0 | 1 | 2 | 2 | 2 | 0 | 2 | 2 | 0 | 2 | 16 |
| Yoshida 2015 | 1 | 0 | 0 | 1 | 2 | 1 | 2 | 0 | 2 | 2 | 1 | 1 | 13 |
| Zhang F. 2020 | 2 | 2 | 0 | 1 | 2 | 1 | 2 | 0 | 2 | 2 | 2 | 2 | 18 |
| Zhang J. 2019 | 1 | 0 | 0 | 1 | 2 | 1 | 2 | 0 | 2 | 2 | 1 | 2 | 14 |

**Supplementary Table 2.** Leave-one-out analysis overall survival

| **Study** | **Sig. (2-tailed)** | **Effect Size** | **95% CI** | |
| --- | --- | --- | --- | --- |
| de Jonge, 2016 | 0.02 | 0.78 | 0,63 | 0.96 |
| Kuenstner, 2021 | 0.01 | 0.76 | 0.61 | 0.94 |
| Laude, 2021 | 0.08 | 0.78 | 0.60 | 1.03 |
| McPhail, 2018 | 0.03 | 0.77 | 0.61 | 0.98 |
| Miyaoka, 2022 | 0.01 | 0.76 | 0.61 | 0.94 |
| Petrich, 2014 | 0.08 | 0.76 | 0.56 | 1.03 |
| Schieppati, 2020 | 0.03 | 0.78 | 0.63 | 0.98 |
| Tisi, 2019 | 0.03 | 0.79 | 0.64 | 0.98 |
| Yoshida, 2015 | 0.02 | 0.77 | 0.62 | 0.96 |
| Zhang, J., 2020 | 0.01 | 0.76 | 0,62 | 0.94 |
| Zhang, F., 2019 | 0.16 | 0.85 | 0,67 | 1.07 |

Sig: significance; CI: confidence interval

**Supplementary Table 3.** Leave-one-out analysis progression-free survival

| **Study** | **Sig. (2-tailed)** | **Effect Size** | **95% CI** | |
| --- | --- | --- | --- | --- |
| Kuenstner, 2021 | <0.05 | 0.57 | 0.45 | 0.71 |
| Laude, 2021 | 0.13 | 0.67 | 0.40 | 1.18 |
| McPhail, 2018 | 0.11 | 0.67 | 0.41 | 1.10 |
| Petrich, 2014 | 0.15 | 0.68 | 0.41 | 1.14 |
| Schieppati, 2020 | 0.11 | 0.70 | 0.46 | 1.08 |
| Tisi, 2019 | 0.07 | 0.66 | 0.41 | 1.05 |
| Zhang, F., 2019 | 0.15 | 0.74 | 0.49 | 1.12 |

Sig: significance; CI: confidence interval

**Supplementary Figure 1**. Funnel plot OS


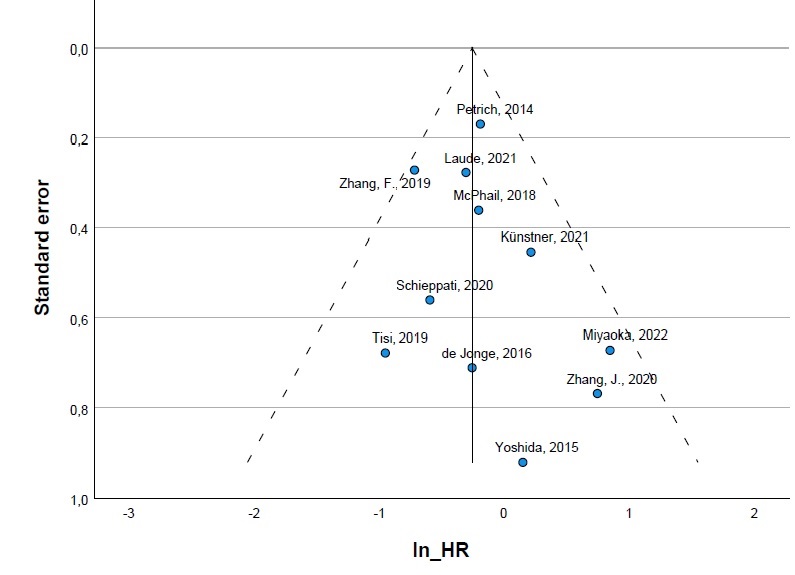


**Supplementary Figure 2.** Funnel plot PFS


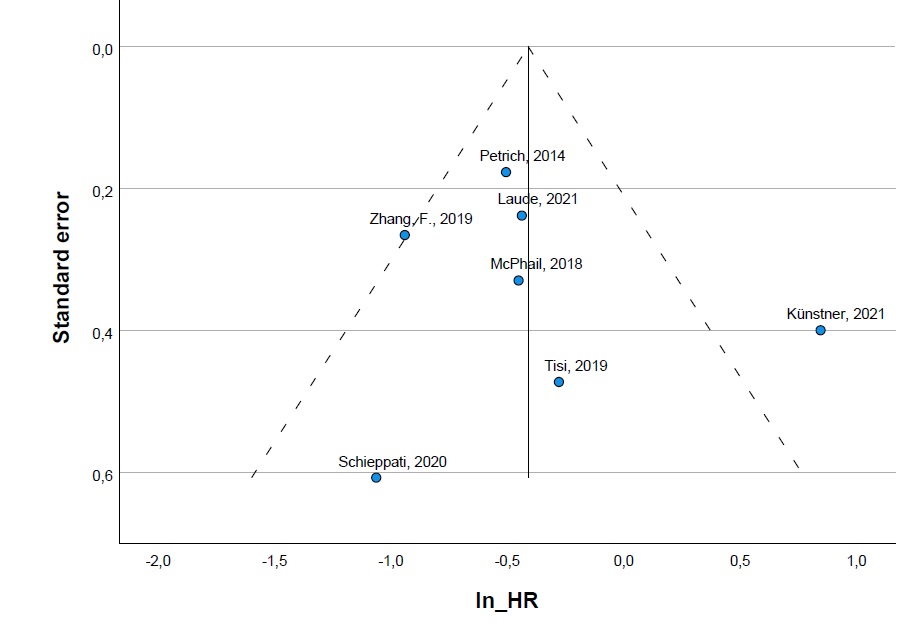

Supplement: Supplementary file 1 [file DataSheet_1.docx]
